# Association between omega-3/6 fatty acids and cholelithiasis: A mendelian randomization study

**DOI:** 10.3389/fnut.2022.964805

**Published:** 2022-09-23

**Authors:** Qi Sun, Ning Gao, Weiliang Xia

**Affiliations:** ^1^Division of Hepatobiliary and Pancreatic Surgery, Department of Surgery, First Affiliated Hospital, School of Medicine, Zhejiang University, Hangzhou, China; ^2^Key Laboratory of Combined Multi-organ Transplantation, Ministry of Public Health, First Affiliated Hospital, School of Medicine, Zhejiang University, Hangzhou, China; ^3^Department of Cardiovascular Surgery, The Second Affiliated Hospital, School of Medicine, Zhejiang University, Hangzhou, China

**Keywords:** cholelithiasis, polyunsaturated acids, omega-3 (ω-3) and omega-6 (ω-6) fatty acids, mendelian randomisation, causal relationship

## Abstract

**Background:**

Omega-3 and omega-6 may be protective factors for cholelithiasis. However, this relationship has not yet been demonstrated clearly. Therefore, we attempted to identify these causal relationships.

**Materials and methods:**

The omega-3/6 fatty acid discovery dataset was obtained from UK Biobank and contained 114,999 individuals. The validation set was derived from an independent genome-wide association study (GWAS) and contained 13,544 individuals. The cholelithiasis dataset was derived from FinnGen and contained 19,023 cases and 195,144 controls. The inverse variance weighting (IVW) method was used as the main method of analysis in this study. Multiple methods of analysis were also used in the repeated methods, including the MR-Egger, weighted median, MR-pleiotropic residual sum (MR-PRESSO), outliers, and maximum likelihood methods. In addition, we used multiple sensitivity analyses to identify the potential pleiotropy.

**Result:**

In the discovery stage, the results of the random effect IVW analysis showed that higher omega-3 levels were correlated inversely with the risk of cholelithiasis (β = –0.22, 95% CI [–0.32 to –0.12], *P* = 1.49 × 10^–5^). When the replication analysis was performed using another set of instrumental variables (IVs), the causal relationship between omega-3 fatty acids and cholelithiasis remained stable (β = –0.42, 95% CI [–0.66 to –0.18], *P* = 5.49 × 10^–4^), except for the results obtained using the MR-Egger method, which were not significant. The results of the IVW approach showed that each SD increase in omega-6 levels was associated negatively with the risk of cholelithiasis, both in the discovery (β = –0.21, 95% CI [–0.35 to –0.06], *P* = 4.37 × 10^–3^) and the validation phases (β = –0.21, 95% CI [–0.40 to –0.02], *P* = 3.44 × 10^–2^).

**Conclusion:**

The results of our MR study suggest that omega-3/6 is associated with cholelithiasis risk. Attention to the risk of cholelithiasis in individuals with low serum omega-3/6 levels is necessary.

## Introduction

Cholelithiasis is an increasingly common hepatobiliary disease. Approximately 10–20% of adults have had cholelithiasis (1, 2). In addition to biliary malignancy, cholelithiasis is associated strongly with small intestinal, prostate, and kidney cancers (3). This is a public health concern on which greater emphasis should be placed.

In general, cholelithiasis can be classified as cholesterol and pigment gallstones according to the composition, with cholesterol gallstones accounting for approximately 80–90% of all the gallstones in most western countries (1, 4). Hepatic cholesterol hypersecretion, supersaturated bile juice, and gallbladder hypomotility contribute to the pathophysiology of cholesterol gallstones. These factors work collaboratively and cause the failure of biliary cholesterol solubility homeostasis, which subsequently results in cholesterol crystallization in bile juice and eventually biliary stone formation (1).

Among the polyunsaturated fatty acids (PUFAs), omega-3 (ω-3) and omega-6 (ω-6) are the two main families that have been shown to be relevant to human health (5, 6). In animal studies, it has been confirmed that high intake of PUFAs can decrease the risk of cholelithiasis by reducing the cholesterol saturation index (CSI) and suppressing the production of gallbladder mucin which is regarded as a trigger for gallstone formation (7, 8). Furthermore, it was reported that PUFAs combined with ursodeoxycholic acid can dissolve cholesterol stones in mice (9). However, the beneficial effects of PUFAs in humans remain debatable. While a prospective cohort study linked high intakes to a reduced prevalence of cholelithiasis in men (4), an epidemiologic study demonstrated that PUFA intake had no effect on cholelithiasis development (10).

Therefore, it is necessary to understand the causal relationship between PUFAs and cholelithiasis. Mendelian randomisation (MR) is an emerging epidemiological method that uses genetic variation as an instrumental variable (IV) to assess the causal association between exposure and outcome (11). Genetic variation is passed randomly to offspring during meiosis, and thus, its estimates of causal effects are consistent with the time order in which they should be. More importantly, the use of MR minimizes the interference of confounding variables between exposure and outcome by avoiding confounding factors to the greatest extent possible (12). Therefore, to examine the potential causal relationship between PUFAs and cholelithiasis, we performed an MR analysis of two samples using summary-level genome-wide association study (GWAS) data and validated them using additional datasets.

## Materials and methods

### Study design

**FIGURE 1:**
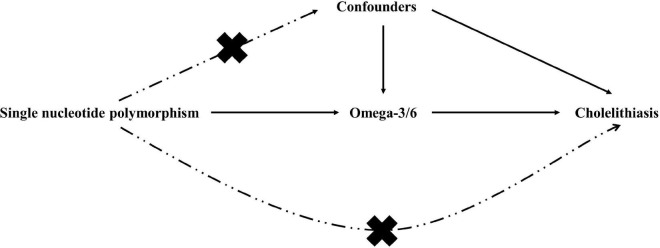
Basic assumptions of mendelian randomization.

**FIGURE 2:**
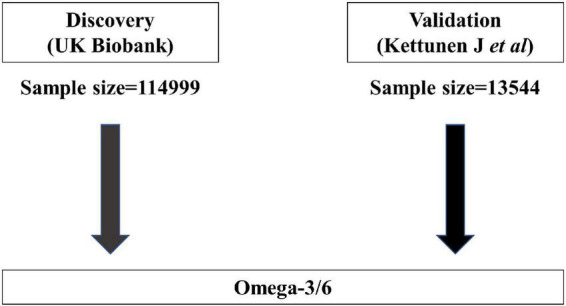
Main design of this study.

Similar to most MR analyses, our study rested on the following three assumptions: genetic variation is linked strongly to exposure, genetic variants should not be considered confounders, and genetic variants should be related to outcomes only *via* exposure (13). We used two exposure datasets from different sources for the analysis: the discovery and validation sets. Figures 1, 2 show the overview of the study’s design. Ethical review approval and informed consent were obtained for the original study.

### Selection of instrumental variables

The genetic instrumental variables for ω-3/6 were derived from the UK Biobank (UKB) and included 114,999 participants (Table 1). The data were adjusted for age, age squared, and sex. Since this GWAS study included more participants and analyzed more single nucleotide polymorphisms (SNPs), it was used as the discovery set. We screened for SNPs with genome-wide significance (*P* < 5 × 10^–8^). Subsequently, to ensure that the SNPs were valid and independent, we removed the linkage disequilibrium (LD) between the SNPs at *r*^2^ < 0.001. Furthermore, the secondary phenotype of each SNP was retrieved to ensure that it was not associated with cholelithiasis. The F-statistic was performed to assess the strength of the IVs. The threshold of the F statistic > 10 indicated a relatively strong estimated effect of IVs (14).

**TABLE 1:** Demographic overview and strength assessment.

Traits	Data sources	Sample size (case/control)	Ancestry	*R*^2^ (%)	*F-*statistic (total)
**Exposure**					
Omega-3 Discovery	UK Biobank	114,999	European	4.10	103.15
Omega-6 Discovery	UK Biobank	114,999	European	0.98	26.70
Omega-3 Validation	Kettunen J. et al.	13,544	European	1.55	40.11
Omega-6 Validation	Kettunen J. et al.	13,544	European	1.08	16.43
**Outcome**					
Cholelithiasis	FinnGen	19,023/195,144	European		

Another group of ω-3/6 IVs was derived from a GWAS containing 13,544 European participants (15). The screening criteria were the same as those described previously. This set of IVs was used as the validation set.

### Outcome data source

GWAS summary statistics for gallstone disease were obtained from the FinnGen Consortium^1^ (Table 1). This was a large cohort study analyzing more than 16,000,000 SNPs, adjusted for sex, age, and genotyping batches. The definition of cholelithiasis in this study was based on the K80 type in ICD-10, and strict SNP inclusion criteria (MAF > 1%) were used. Including 19,023 cholelithiasis cases and 195,144 controls were included in this study.

### Statistical analysis

The random-effects model inverse variance weighting method was used as the main method of analysis in this study (16). Multiple analysis methods have also been used for repeated analysis, including the MR-Egger (17), weighted median (18), MR-pleiotropic residual sum and outliers (19) and maximum likelihood methods (20). Each approach employs different hypothetical models to assess causal effects, which are then used to check the robustness of the results. The MR-Egger provides calculation after adjusting for pleiotropy (17). Median weighting allowed estimation of causal effects when 50% of SNPs were invalid (18). The median weighting method allows for the estimation of causal effects when 50% of the SNPs are invalid (18). The MR-PRESSO method detects and corrects outliers, providing MR calculation that are robust in terms of heterogeneity after removing the identified outliers (19).

An MR analysis is often confounded by horizontal pleiotropy, which can lead to biased results. Therefore, we used multiple sensitivity analyses to identify potential pleiotropy. First, Cochran’s Q statistic was used to assess heterogeneity among the SNPs. Cochran’s Q-derived *P* < 0.05 was considered an indicator of heterogeneity in the IVs, at which point the multiplicative random effects IVW method was considered the gold standard (17). Second, an intercept test of the MR-Egger method was performed to measure horizontal multiplicity (17). Third, a leave-one-out analysis was performed to assess whether the association was driven by a single SNP (17).

The correlations with a *P*-value < 0.05 were considered to be statistically significant. All the analyses were performed using R software (version 4.1.2). The MR analyses were performed using the R packages “TwoSampleMR” and “MendelianRandomization.”

## Results

The instrumental variable strength analysis showed that the general F statistic was greater than the empirical threshold of 10 (Table 1), indicating that a weak instrumental bias was unlikely to affect the estimation of the causal effects. Using PhenoScanner 2, we did not find any IVs of ω-3/6 that were associated with potential confounding factors.

### Genetic liability to omega-3 with cholelithiasis

**FIGURE 3:**
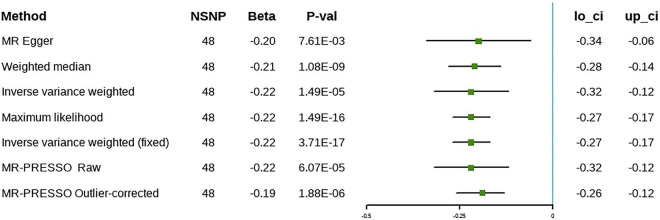
The causal relationship between omega-3 and cholelithiasis (discovery).

In the discovery stage, the random effect IVW analysis showed that higher ω-3 levels were correlated inversely with the risk of cholelithiasis (β = –0.22, 95% CI [–0.32 to –0.12], *P* = 1.49 × 10^–5^) (Figure 3). The Cochran’s Q statistic suggested heterogeneity; therefore, we adopted the results of the random-effect IVW analysis. The MR-Egger intercept method revealed no evidence of horizontal pleiotropy (Table 2). The remaining analyses showed that the significant results were not driven by any single SNP (Supplementary Figure 3). The MR-Egger, weighted median, and maximum likelihood methods produced the same results as the IVW method (Figure 3). The MR-PRESSO method detected outliers; however, the results did not change after the outliers were removed, which further demonstrated the reliability of our results. Forest plots and funnel plots are presented in Supplementary Figures 2, 3. Detailed information on the SNPs involved is in Supplementary Table 1.

**TABLE 2:** Pleiotropy and heterogeneity test of the omega-3/6 IVs from cholelithiasis GWAS.

		Pleiotropy test			Heterogeneity test			
		
		MR-Egger			MR-Egger		IVW	
			
		Intercept	SE	P	Q	Q_pval	Q	Q_pval
Omega-3	Discovery	–0.003	0.006	6.78E-01	177.075	2.86E-17	177.746	4.42E-17
	Validation	–0.025	0.059	6.95E-01	19.572	2.08E-04	20.788	3.49E-04
Omega-6	Discovery	0.001	0.009	8.87E-01	147.322	2.41E-13	147.392	4.41E-13
	Validation	–0.030	0.037	4.52E-01	43.603	2.55E-07	47.555	1.20E-07

**FIGURE 4:**
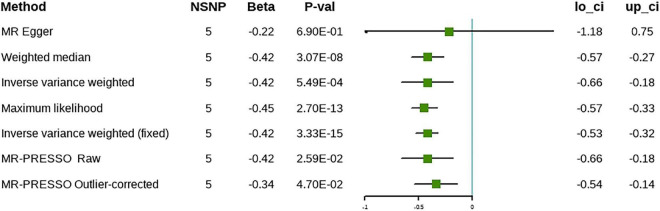
The causal relationship between omega-3 and cholelithiasis (validation).

When the replication analysis was performed using another set of IVs, the causal relationship between ω-3 and cholelithiasis remained stable (β = –0.42, 95% CI [–0.66 to –0.18], *P* = 5.49 × 10^–4^), except for the findings that were obtained using the MR-Egger method, which were not significant (Figure 4). Further sensitivity analyses showed no evidence of horizontal pleiotropy despite the heterogeneity of the IVs. The results of the leave-one-out analysis were consistent with the discovery phase and the results were not caused by any single SNP (Supplementary Figure 3). The results of the MR-PRESSO method remained significant after removal of the outliers. Forest plots and funnel plots are presented in Supplementary Figures 2, 3. Detailed information on the SNPs involved is in Supplementary Table 2.

### Genetic liability to omega-6 with cholelithiasis

**FIGURE 5:**
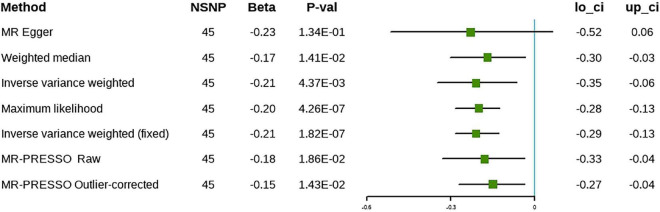
The causal relationship between omega-6 and cholelithiasis (discovery).

**FIGURE 6:**
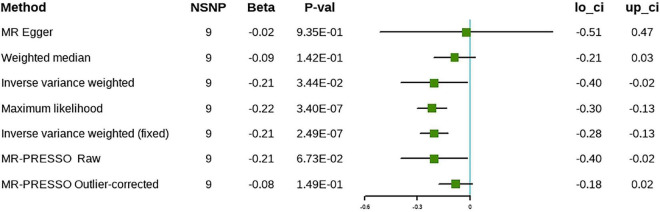
The causal relationship between omega-6 and cholelithiasis (validation).

The results of the IVW approach showed that each SD increase in ω-6 was associated negatively with the risk of cholelithiasis, both in the discovery (β = –0.21, 95% CI [–0.35 to –0.06], *P* = 4.37 × 10^–3^) and in the validation phases (β = –0.21, 95% CI [–0.40 to –0.02], *P* = 3.44 × 10^–2^) (Figures 5, 6). The sensitivity analysis showed no evidence of horizontal pleiotropy, although heterogeneity was observed among the IVs (Table 2). Furthermore, the ω-6 association with cholelithiasis was not driven by any SNP (Supplementary Figure 3). In the discovery phase, the results of the MR-PRESSO method were consistent with the original values, after the removal of the outliers. However, the results were inconsistent after removing the outliers during the validation phase. One possible explanation is the heterogeneity of the IVs. However, since the validation set contained fewer IVs, the excessive elimination of SNPs would result in a loss of statistical power. Forest plots and funnel plots are presented in Supplementary Figures 2, 3. Detailed information on the SNPs involved is in Supplementary Tables 3, 4.

## Discussion

In this study, both the discovery phase and the validation phase suggest that higher serum omega-3/6 fatty acid concentrations may be associated with a lower risk of cholelithiasis. At the same time, sensitivity analysis found that horizontal pleiotropy did not significantly interfere with the results of this study.

Epidemiological or clinical studies on the relationship between PUFA consumption and the risk of cholelithiasis have been conflicting and sparse. In a prospective cohort study, the high consumption of PUFAs was correlated inversely with the risk of cholelithiasis in men (4). This was supported by a cross-sectional study which indicated that high consumption of PUFAs played a protective role in cholelithiasis (21). However, PUFA intake did not seem to be associated with cholelithiasis in a observational study that was conducted in Argentina (22). Furthermore, the intake of PUFAs in patients with gallstones was higher in a retrospective study that was conducted in Spain (23). These results may have been due to the use of small sample sizes or the lack of long-term dietary information.

Our results may be explained by several possible underlying mechanisms. It has been reported previously that ω-3 PUFA supplementation may modify the composition of biliary phosphatidylcholine (24). This change may stabilize the cholesterol-phospholipid vesicles, which play a significant role in preventing cholesterol nucleation and gallstone formation (25). The reason for this change may be the fact that supplementation with ω-3 PUFA down-regulates the expression of canalicular transporters ABC, which has a major role in cholesterol secretion. Second, the antinucleating effect of ω-3 PUFA may also be explained by the arachidonic acid (AA) hypothesis. According to a study on the African Green Monkey, a high intake of ω-3 PUFA may reduce the percentage of AA in biliary phospholipids (26). In addition, the presence of AA in biliary phospholipids causes the hypersecretion of gallbladder mucins which has been considered to trigger the formation of gallstones by serving as a nidus of gallstones (27, 28). Besides, a high intake of ω-3 PUFA can also decrease mucins secretion by reducing expression of mucin gene expression such as Muc2, Muc5ac, Muc5b. Third, previous studies have demonstrated that dietary supplementation with ω-3 PUFA promoted the secretion of hepatic phospholipids by reducing the hydrophobicity of phospholipids (26, 29). Enhanced hepatic phospholipid secretion may increase the bile phospholipid concentration and reduce CSI. Finally, the effects of ω-3 PUFA may also be explained by increased insulin sensitivity. Metabolic studies have suggested that an increased intake of ω-3 PUFA may improve insulin sensitivity by changing the fatty acid composition of the adipocyte plasma membrane (30, 31). In addition, there is an increased synthesis of cholesterol and hypersecretion of biliary cholesterol in patients with insulin resistance (32–34). Previous studies have also speculated that insulin resistance may participate in the pathogenesis of cholelithiasis by promoting the release of proinflammatory cytokines that are related to gallbladder inflammation (35, 36).

Recent evidence has shown that ω-6 PUFA is inversely associated with type 2 diabetes mellitus (T2DM) (37). T2DM has been proved to be a high-risk factor for cholelithiasis. Furthermore, ω-6 PUFA may significantly decrease triglycerides and increase high-density lipoprotein (HDL) cholesterol levels (38). High triglyceride and low HDL cholesterol levels are established risk factors for cholelithiasis. In addition, ω-6 PUFA can promote the production and secretion of bile acids by inducing the synthesis of cholesterol 7α-hydroxylase (39). This may be related to the reduced expression of sterol 27-hydroxylase. Therefore, this suggested that ω-6 PUFA may also reduce CSI and prevent cholelithiasis.

Although laparoscopic cholecystectomy has become the most common minimally surgical procedure performed worldwide, it may be suboptimal in the long term (9, 40). As a surgical procedure, laparoscopic cholecystectomy is inevitably associated with surgical complications and even patient death (41, 42). In addition, cholelithiasis is considered to be one of the highest medical burdens among digestive diseases. In the future, more attention should be paid to preventing cholelithiasis. Our study may promote a paradigm shift from the diagnosis and treatment of gallstones to prevention. Patients with a strong susceptibility to gallstones may benefit from preventive PUFA supplementation.

Our study had several strengths. Firstly, for the first time, the causal association between omega fatty acids and cholelithiasis was explored using MR analysis; secondly, this study consisted of two parts: discovery and validation, which made the results more reliable, and there was no overlap in the population between different data sets. Thirdly, a series of replicate and sensitivity analyses were applied to improve the credibility of our results.

Our study also had several limitations. First, the participants involved in this study were of European origin; therefore, this result should be interpreted with caution in other populations. Second, there was heterogeneity among the IVs used in this study; however, the absence of pleiotropy in the MR-Egger test suggested balanced pleiotropy, which was unlikely to bias the results. Third, although we used several approaches to remove confounders and minimize the possibility of bias, the potential pleiotropy could not be removed completely. However, the sensitivity analyses suggested that horizontal pleiotropy was unlikely to have an impact on our results.

## Conclusion

In summary, our findings indicate that individuals with lower omega-3/6 fatty acid levels have a higher risk of cholelithiasis. Given the greater disease burden of cholelithiasis, ω-3/6 fatty acid supplementation may be a promising adjunct treatment modality. Standardized randomized controlled trials should be designed to further explore the benefits of PUFAs in cholelithiasis.

## Data availability statement

The original contributions presented in this study are included in the article/Supplementary material, further inquiries can be directed to the corresponding author.

## Author contributions

QS and WX designed the study and drafted the article. QS, NG, and WX conducted the data acquisition and performed the data analysis and manuscript revision. All authors have read and approved the final manuscript.

## Footnotes

1
https://r5.finngen.fi/
