# Supplementary material for: Association between omega-3/6 fatty acids and cholelithiasis: A mendelian randomization study
Source: Front Nutr. 2022 Sep 23;9:964805. doi: 10.3389/fnut.2022.964805 (PMC9537577; doi:10.3389/fnut.2022.964805)
Supplement: Supplementary file 1 [file Data_Sheet_1.docx]

Supplementary Material

# Supplementary Figures and Tables

## Supplementary Figures


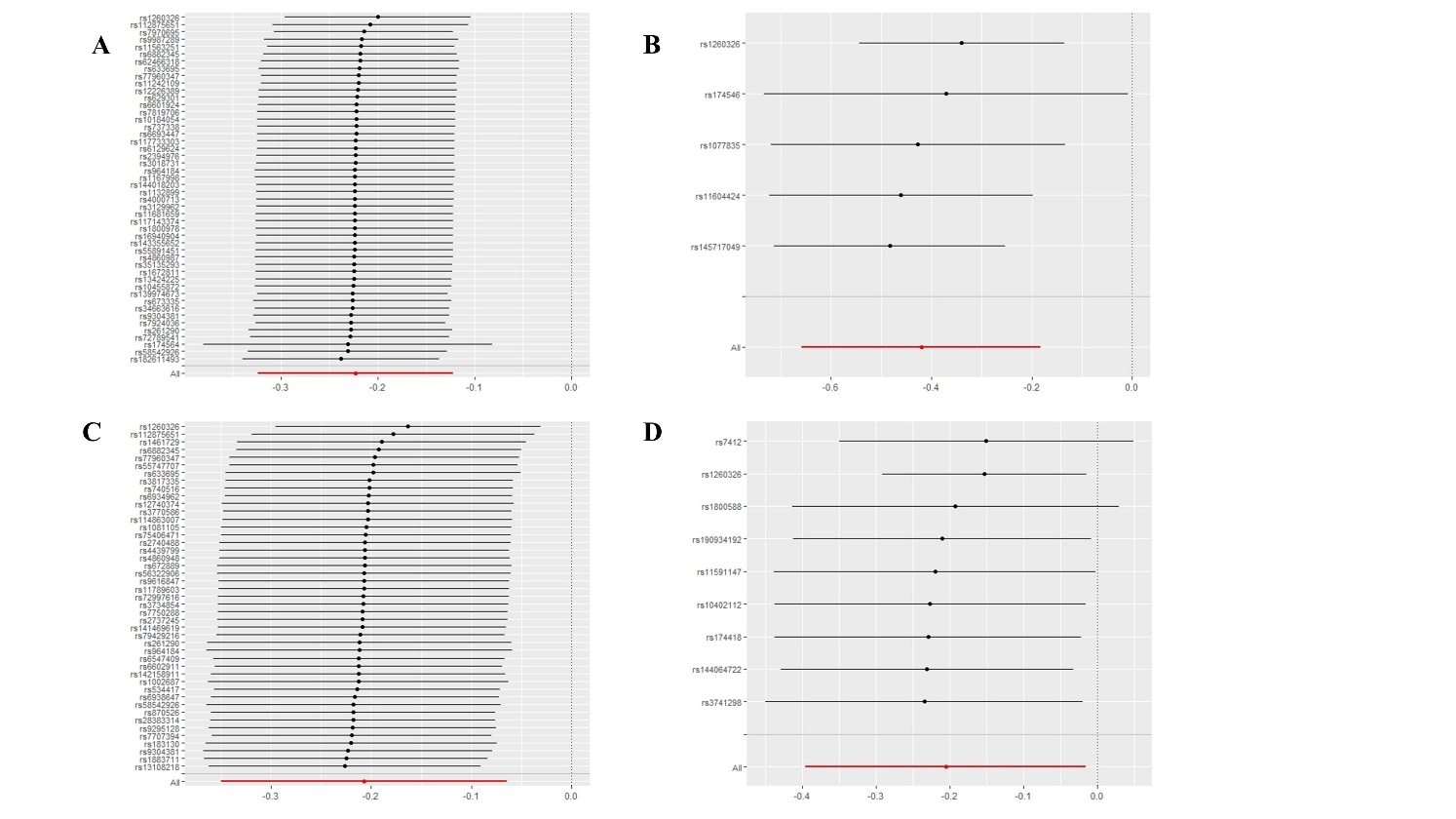


**Supplementary Figure 1.** Leave-one-out: (A) Omega-3 (discovery) and Cholelithiasis; (B) Omega-3 (discovery) and Cholelithiasis; (C) Psoriasis and Myocardial infarction; (D) Psoriasis and Myocardial infarction;

##
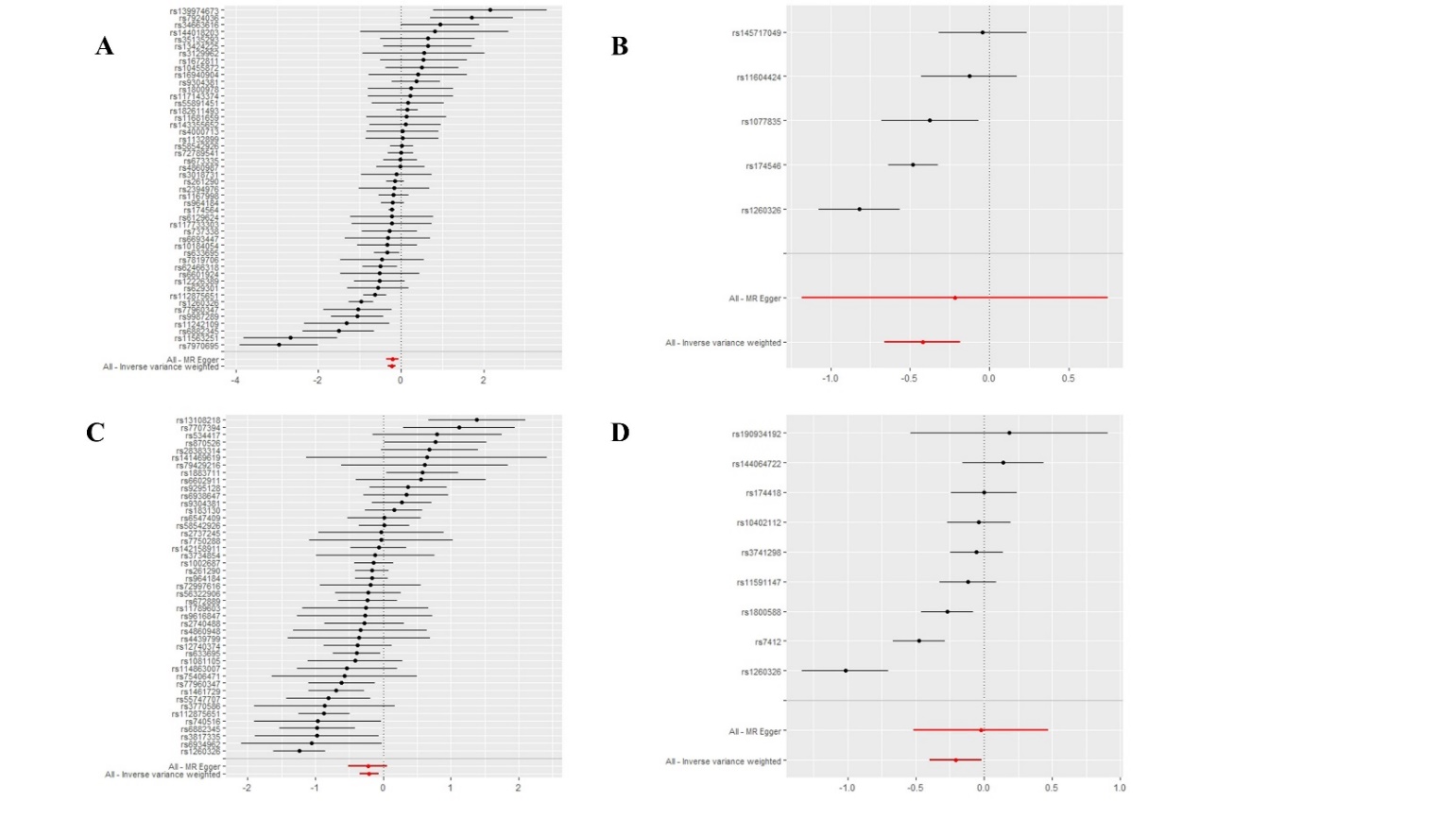
Supplementary Figure 2. Forest plot: (A) Omega-3 (discovery) and Cholelithiasis; (B) Omega-3 (discovery) and Cholelithiasis; (C) Psoriasis and Myocardial infarction; (D) Psoriasis and Myocardial infarction;


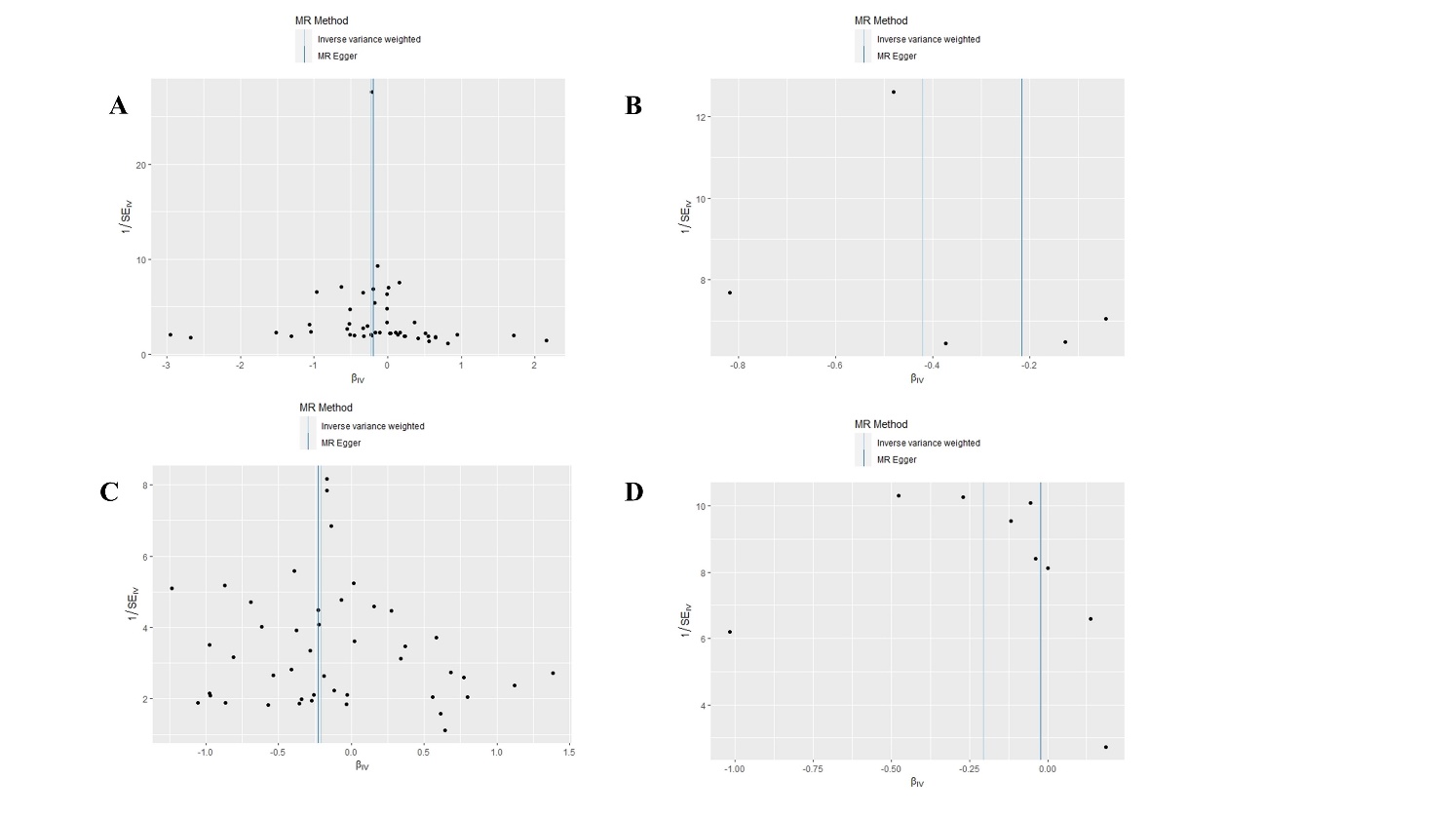


**Supplementary Figure 3.** Funnel plot: (A) Omega-3 (discovery) and Cholelithiasis; (B) Omega-3 (discovery) and Cholelithiasis; (C) Psoriasis and Myocardial infarction; (D) Psoriasis and Myocardial infarction;

## Supplementary Tables

Supplementary Table 1 Summary information on the SNPs used as genetic instruments for the omega-3(Discovery) in MR study

| SNP | chr | pos | A1 | A2 | eaf.exposure | Exporsure(Omega-3) | | | Outcome(Cholelithiasis) | | |
| --- | --- | --- | --- | --- | --- | --- | --- | --- | --- | --- | --- |
|  |  |  |  |  |  | β | Se | *P* | β | Se | *P* |
| rs10184054 | 2 | 21203877 | G | C | 0.224107 | -0.036 | 0.005 | 5.60E-15 | 0.012 | 0.013 | 3.69E-01 |
| rs10455872 | 6 | 161010118 | G | A | 0.078988 | -0.063 | 0.008 | 2.80E-17 | -0.032 | 0.029 | 2.58E-01 |
| rs11242109 | 5 | 131677047 | T | G | 0.479016 | 0.024 | 0.004 | 2.40E-09 | -0.032 | 0.013 | 1.25E-02 |
| rs112875651 | 8 | 126506694 | A | G | 0.392346 | -0.087 | 0.004 | 3.50E-98 | 0.056 | 0.012 | 6.76E-06 |
| rs1132899 | 19 | 45448036 | C | T | 0.509603 | 0.027 | 0.004 | 8.60E-11 | 0.001 | 0.012 | 9.48E-01 |
| rs11563251 | 2 | 234679384 | T | C | 0.110601 | 0.035 | 0.006 | 3.20E-08 | -0.094 | 0.020 | 3.58E-06 |
| rs1167998 | 1 | 62931632 | A | C | 0.644674 | 0.071 | 0.004 | 3.60E-66 | -0.013 | 0.013 | 3.32E-01 |
| rs11681659 | 2 | 136820960 | T | C | 0.716465 | -0.025 | 0.004 | 2.00E-08 | -0.004 | 0.012 | 7.81E-01 |
| rs117143374 | 21 | 40555561 | C | T | 0.142254 | -0.037 | 0.006 | 2.20E-10 | -0.008 | 0.020 | 6.69E-01 |
| rs117733303 | 6 | 160922870 | G | A | 0.018513 | -0.116 | 0.015 | 1.40E-15 | 0.026 | 0.057 | 6.50E-01 |
| rs12226389 | 11 | 61823630 | C | T | 0.18582 | -0.051 | 0.005 | 1.10E-22 | 0.027 | 0.016 | 9.23E-02 |
| rs1260326 | 2 | 27730940 | C | T | 0.60401 | -0.082 | 0.004 | 8.40E-88 | 0.079 | 0.013 | 3.46E-10 |
| rs13424225 | 2 | 241214158 | T | G | 0.449809 | 0.022 | 0.004 | 2.20E-08 | 0.014 | 0.012 | 2.33E-01 |
| rs139974673 | 15 | 44027885 | C | T | 0.025918 | 0.118 | 0.013 | 2.30E-21 | 0.254 | 0.083 | 2.20E-03 |
| rs143355652 | 11 | 61453822 | T | C | 0.010467 | -0.154 | 0.020 | 9.40E-14 | -0.017 | 0.068 | 8.06E-01 |
| rs144018203 | 11 | 116916060 | C | G | 0.010653 | 0.107 | 0.020 | 4.20E-08 | 0.087 | 0.098 | 3.72E-01 |
| rs1672811 | 16 | 15501099 | C | T | 0.748488 | 0.025 | 0.005 | 3.00E-08 | 0.014 | 0.013 | 3.03E-01 |
| rs16940904 | 17 | 44186063 | T | C | 0.226571 | -0.036 | 0.005 | 3.90E-14 | -0.015 | 0.022 | 5.00E-01 |
| rs174564 | 11 | 61588305 | G | A | 0.347013 | -0.337 | 0.004 | 1.00E-200 | 0.073 | 0.012 | 2.22E-09 |
| rs1800978 | 9 | 107665978 | G | C | 0.123991 | -0.037 | 0.006 | 5.20E-09 | -0.009 | 0.020 | 6.53E-01 |
| rs182611493 | 19 | 19458388 | G | A | 0.012519 | -0.210 | 0.020 | 1.10E-27 | -0.033 | 0.028 | 2.45E-01 |
| rs2394976 | 6 | 31311912 | T | G | 0.161648 | -0.046 | 0.006 | 1.20E-15 | 0.008 | 0.020 | 6.96E-01 |
| rs261290 | 15 | 58678720 | C | T | 0.654653 | -0.114 | 0.004 | 3.90E-161 | 0.016 | 0.012 | 1.89E-01 |
| rs3018731 | 11 | 61248776 | G | A | 0.717549 | -0.035 | 0.005 | 2.00E-14 | 0.004 | 0.015 | 8.05E-01 |
| rs3129962 | 6 | 32379383 | A | G | 0.129765 | -0.039 | 0.006 | 1.80E-09 | -0.022 | 0.030 | 4.61E-01 |
| rs34663616 | 15 | 58569330 | A | C | 0.137654 | 0.036 | 0.006 | 4.40E-10 | 0.034 | 0.017 | 4.94E-02 |
| rs35135293 | 2 | 20363666 | T | C | 0.51675 | -0.021 | 0.004 | 3.90E-08 | -0.014 | 0.012 | 2.67E-01 |
| rs4000713 | 7 | 25990597 | A | G | 0.295408 | -0.029 | 0.004 | 1.00E-11 | -0.001 | 0.013 | 9.29E-01 |
| rs4860987 | 4 | 69491284 | T | A | 0.258609 | 0.046 | 0.005 | 1.20E-21 | -0.001 | 0.014 | 9.58E-01 |
| rs55891451 | 10 | 96728169 | C | A | 0.201728 | 0.034 | 0.005 | 4.60E-12 | 0.006 | 0.015 | 7.04E-01 |
| rs58542926 | 19 | 19379549 | T | C | 0.074383 | -0.172 | 0.008 | 1.40E-113 | -0.002 | 0.024 | 9.33E-01 |
| rs6129624 | 20 | 39167592 | A | G | 0.335237 | -0.026 | 0.004 | 5.10E-10 | 0.006 | 0.013 | 6.69E-01 |
| rs62466318 | 7 | 73042085 | T | C | 0.204178 | -0.072 | 0.005 | 1.20E-45 | 0.037 | 0.015 | 1.58E-02 |
| rs629301 | 1 | 109818306 | T | G | 0.778033 | 0.038 | 0.005 | 1.30E-14 | -0.021 | 0.015 | 1.50E-01 |
| rs633695 | 15 | 58725839 | G | A | 0.292348 | 0.084 | 0.004 | 9.10E-80 | -0.029 | 0.013 | 2.88E-02 |
| rs6601924 | 10 | 5247302 | C | T | 0.845765 | 0.035 | 0.006 | 8.50E-10 | -0.018 | 0.017 | 2.97E-01 |
| rs6693447 | 1 | 2330190 | G | T | 0.461686 | 0.023 | 0.004 | 4.80E-09 | -0.007 | 0.012 | 5.41E-01 |
| rs673335 | 11 | 75450576 | C | T | 0.159762 | -0.067 | 0.006 | 1.10E-34 | 0.001 | 0.014 | 9.56E-01 |
| rs6882345 | 5 | 156397673 | A | G | 0.632863 | 0.029 | 0.004 | 1.90E-13 | -0.044 | 0.013 | 6.10E-04 |
| rs72789541 | 16 | 15127534 | A | T | 0.29597 | -0.081 | 0.004 | 5.60E-75 | 0.001 | 0.013 | 9.67E-01 |
| rs737338 | 19 | 11347657 | T | C | 0.035186 | -0.073 | 0.011 | 3.50E-11 | 0.020 | 0.025 | 4.10E-01 |
| rs77960347 | 18 | 47109955 | G | A | 0.013239 | 0.162 | 0.018 | 7.20E-22 | -0.169 | 0.069 | 1.38E-02 |
| rs7819706 | 8 | 19844415 | G | A | 0.118291 | -0.040 | 0.006 | 1.80E-10 | 0.018 | 0.021 | 3.77E-01 |
| rs7924036 | 10 | 65191645 | T | G | 0.504205 | 0.023 | 0.004 | 5.50E-10 | 0.040 | 0.012 | 8.44E-04 |
| rs7970695 | 12 | 121423376 | A | G | 0.620549 | -0.025 | 0.004 | 1.20E-10 | 0.075 | 0.012 | 9.97E-10 |
| rs9304381 | 18 | 47158234 | T | C | 0.818434 | 0.053 | 0.005 | 5.20E-24 | 0.019 | 0.016 | 2.17E-01 |
| rs964184 | 11 | 116648917 | C | G | 0.867229 | -0.117 | 0.006 | 8.90E-87 | 0.024 | 0.017 | 1.67E-01 |
| rs9987289 | 8 | 9183358 | G | A | 0.909151 | 0.057 | 0.007 | 3.20E-16 | -0.060 | 0.018 | 9.79E-04 |

Supplementary Table 2 Summary information on the SNPs used as genetic instruments for the omega-3(Validation) in MR study

| SNP | chr | pos | A1 | A2 | eaf.exposure | Exporsure(Omega-3) | | | Outcome(Cholelithiasis) | | |
| --- | --- | --- | --- | --- | --- | --- | --- | --- | --- | --- | --- |
|  |  |  |  |  |  | β | Se | *P* | β | Se | *P* |
| rs1077835 | 15 | 58723426 | G | A | 0.250 | 0.089 | 0.014 | 1.08E-09 | -0.033 | 0.014 | 1.60E-02 |
| rs11604424 | 11 | 116651115 | T | C | 0.756 | -0.090 | 0.014 | 3.32E-10 | 0.011 | 0.014 | 4.15E-01 |
| rs1260326 | 2 | 27730940 | C | T | 0.637 | -0.097 | 0.013 | 3.37E-14 | 0.079 | 0.013 | 3.46E-10 |
| rs145717049 | 19 | 19130096 | T | C | 0.044 | -0.191 | 0.033 | 6.67E-09 | 0.008 | 0.027 | 7.66E-01 |
| rs174546 | 11 | 61569830 | T | C | 0.403 | -0.154 | 0.012 | 1.19E-34 | 0.074 | 0.012 | 1.26E-09 |

Supplementary Table 3 Summary information on the SNPs used as genetic instruments for the omega-6(Discovery) in MR study

| SNP | chr | pos | A1 | A2 | eaf.exposure | Exporsure(Omega-6) | | | Outcome(Cholelithiasis) | | |
| --- | --- | --- | --- | --- | --- | --- | --- | --- | --- | --- | --- |
|  |  |  |  |  |  | β | Se | *P* | β | Se | *P* |
| rs1002687 | 1 | 62963737 | A | G | 0.645 | 0.091 | 0.004 | 1.000E-107 | -0.013 | 0.013 | 3.355E-01 |
| rs1081105 | 19 | 45412955 | C | A | 0.028 | 0.119 | 0.012 | 1.800E-22 | -0.049 | 0.042 | 2.438E-01 |
| rs112875651 | 8 | 126506694 | A | G | 0.392 | -0.064 | 0.004 | 2.200E-53 | 0.056 | 0.012 | 6.760E-06 |
| rs114863007 | 6 | 34729158 | A | G | 0.095 | -0.046 | 0.007 | 7.300E-12 | 0.025 | 0.017 | 1.548E-01 |
| rs11789603 | 9 | 107647019 | T | C | 0.109 | 0.048 | 0.006 | 9.700E-14 | -0.012 | 0.023 | 5.847E-01 |
| rs1260326 | 2 | 27730940 | C | T | 0.604 | -0.064 | 0.004 | 3.900E-55 | 0.079 | 0.013 | 3.460E-10 |
| rs12740374 | 1 | 109817590 | T | G | 0.221 | -0.057 | 0.005 | 1.500E-32 | 0.022 | 0.015 | 1.421E-01 |
| rs13108218 | 4 | 3443931 | G | A | 0.615 | -0.035 | 0.004 | 3.600E-18 | -0.049 | 0.013 | 1.568E-04 |
| rs141469619 | 11 | 116714293 | G | A | 0.010 | 0.111 | 0.021 | 1.400E-08 | 0.071 | 0.100 | 4.776E-01 |
| rs142158911 | 19 | 11190534 | A | G | 0.117 | -0.094 | 0.006 | 5.200E-52 | 0.006 | 0.020 | 7.510E-01 |
| rs1461729 | 8 | 9187242 | G | A | 0.899 | 0.084 | 0.007 | 2.800E-36 | -0.058 | 0.018 | 1.058E-03 |
| rs183130 | 16 | 56991363 | T | C | 0.324 | 0.062 | 0.004 | 1.400E-48 | 0.010 | 0.013 | 4.697E-01 |
| rs1883711 | 20 | 39179822 | C | G | 0.031 | 0.092 | 0.012 | 3.200E-16 | 0.054 | 0.025 | 3.096E-02 |
| rs261290 | 15 | 58678720 | C | T | 0.655 | -0.097 | 0.004 | 1.000E-116 | 0.016 | 0.012 | 1.890E-01 |
| rs2737245 | 8 | 116658583 | T | G | 0.279 | -0.027 | 0.005 | 1.400E-09 | 0.001 | 0.013 | 9.493E-01 |
| rs2740488 | 9 | 107661742 | C | A | 0.265 | -0.050 | 0.005 | 5.400E-28 | 0.014 | 0.015 | 3.482E-01 |
| rs28383314 | 6 | 32587213 | C | T | 0.623 | 0.039 | 0.004 | 1.700E-18 | 0.027 | 0.014 | 6.217E-02 |
| rs3734854 | 6 | 31078836 | A | G | 0.065 | 0.048 | 0.008 | 5.900E-11 | -0.006 | 0.021 | 7.942E-01 |
| rs3770586 | 2 | 169828995 | T | C | 0.484 | -0.023 | 0.004 | 7.100E-09 | 0.020 | 0.012 | 1.023E-01 |
| rs3817335 | 11 | 47643891 | A | T | 0.351 | -0.028 | 0.004 | 9.800E-12 | 0.027 | 0.013 | 3.583E-02 |
| rs4439799 | 17 | 45781599 | T | C | 0.502 | 0.022 | 0.004 | 1.300E-08 | -0.008 | 0.012 | 5.073E-01 |
| rs4860948 | 4 | 69340991 | A | T | 0.244 | 0.028 | 0.005 | 1.900E-09 | -0.010 | 0.014 | 4.993E-01 |
| rs534417 | 1 | 23784965 | G | A | 0.875 | 0.039 | 0.006 | 9.300E-11 | 0.031 | 0.019 | 1.012E-01 |
| rs55747707 | 7 | 73037366 | A | G | 0.204 | -0.049 | 0.005 | 1.700E-22 | 0.040 | 0.016 | 1.008E-02 |
| rs56322906 | 19 | 11346155 | A | G | 0.035 | -0.100 | 0.011 | 1.200E-19 | 0.022 | 0.025 | 3.654E-01 |
| rs58542926 | 19 | 19379549 | T | C | 0.074 | -0.128 | 0.008 | 2.500E-65 | -0.002 | 0.024 | 9.328E-01 |
| rs633695 | 15 | 58725839 | G | A | 0.292 | 0.073 | 0.004 | 1.300E-59 | -0.029 | 0.013 | 2.884E-02 |
| rs6547409 | 2 | 21190209 | T | C | 0.051 | -0.081 | 0.009 | 2.400E-20 | -0.002 | 0.023 | 9.442E-01 |
| rs6602911 | 13 | 114547372 | T | C | 0.360 | 0.026 | 0.004 | 1.300E-09 | 0.014 | 0.013 | 2.526E-01 |
| rs672889 | 2 | 21319016 | G | T | 0.860 | 0.076 | 0.006 | 1.300E-41 | -0.017 | 0.017 | 3.047E-01 |
| rs6882345 | 5 | 156397673 | A | G | 0.633 | 0.045 | 0.004 | 1.200E-27 | -0.044 | 0.013 | 6.098E-04 |
| rs6934962 | 6 | 116322349 | T | C | 0.400 | 0.023 | 0.004 | 2.200E-08 | -0.024 | 0.012 | 4.736E-02 |
| rs6938647 | 6 | 160986915 | C | A | 0.782 | -0.048 | 0.005 | 1.900E-23 | -0.016 | 0.015 | 2.867E-01 |
| rs72997616 | 11 | 75474195 | A | C | 0.094 | -0.052 | 0.007 | 1.600E-13 | 0.010 | 0.020 | 6.163E-01 |
| rs740516 | 17 | 67082962 | G | C | 0.151 | -0.032 | 0.006 | 1.400E-08 | 0.031 | 0.015 | 4.228E-02 |
| rs75406471 | 10 | 5257647 | A | G | 0.155 | -0.031 | 0.006 | 2.700E-08 | 0.018 | 0.017 | 2.944E-01 |
| rs7707394 | 5 | 74472939 | A | G | 0.357 | 0.030 | 0.004 | 1.200E-12 | 0.033 | 0.012 | 7.569E-03 |
| rs7750288 | 6 | 160400147 | G | A | 0.285 | 0.025 | 0.004 | 1.300E-08 | -0.001 | 0.014 | 9.533E-01 |
| rs77960347 | 18 | 47109955 | G | A | 0.013 | 0.276 | 0.018 | 2.800E-56 | -0.169 | 0.069 | 1.379E-02 |
| rs79429216 | 19 | 45445517 | A | G | 0.013 | 0.151 | 0.018 | 1.300E-17 | 0.093 | 0.095 | 3.283E-01 |
| rs870526 | 2 | 20369562 | T | C | 0.521 | -0.032 | 0.004 | 7.700E-16 | -0.025 | 0.012 | 4.471E-02 |
| rs9295128 | 6 | 160751531 | T | G | 0.017 | -0.196 | 0.016 | 3.400E-36 | -0.073 | 0.057 | 1.999E-01 |
| rs9304381 | 18 | 47158234 | T | C | 0.818 | 0.070 | 0.005 | 7.200E-42 | 0.019 | 0.016 | 2.166E-01 |
| rs9616847 | 22 | 50868669 | T | A | 0.388 | 0.024 | 0.004 | 1.400E-08 | -0.007 | 0.012 | 5.957E-01 |
| rs964184 | 11 | 116648917 | C | G | 0.867 | -0.139 | 0.006 | 1.100E-125 | 0.024 | 0.017 | 1.672E-01 |

Supplementary Table 4 Summary information on the SNPs used as genetic instruments for the omega-6(Validation) in MR study

| SNP | chr | pos | A1 | A2 | eaf.exposure | Exporsure(Omega-6) | | | Outcome(Cholelithiasis) | | |
| --- | --- | --- | --- | --- | --- | --- | --- | --- | --- | --- | --- |
|  |  |  |  |  |  | β | Se | *P* | β | Se | *P* |
| rs10402112 | 19 | 11191677 | A | T | 0.100 | -0.166 | 0.021 | 3.800E-15 | 0.006 | 0.020 | 7.555E-01 |
| rs11591147 | 1 | 55505647 | T | G | 0.029 | -0.309 | 0.040 | 1.120E-14 | 0.037 | 0.032 | 2.593E-01 |
| rs1260326 | 2 | 27730940 | C | T | 0.637 | -0.078 | 0.013 | 9.730E-10 | 0.079 | 0.013 | 3.460E-10 |
| rs144064722 | 4 | 73406173 | G | A | 0.026 | 0.237 | 0.040 | 2.290E-09 | 0.033 | 0.036 | 3.643E-01 |
| rs174418 | 15 | 58687603 | C | T | 0.562 | -0.098 | 0.013 | 5.990E-15 | 0.000 | 0.012 | 9.939E-01 |
| rs1800588 | 15 | 58723675 | T | C | 0.249 | 0.143 | 0.014 | 9.470E-23 | -0.039 | 0.014 | 5.626E-03 |
| rs190934192 | 1 | 55334001 | A | G | 0.028 | -0.244 | 0.043 | 2.030E-08 | -0.046 | 0.090 | 6.120E-01 |
| rs3741298 | 11 | 116657561 | T | C | 0.770 | -0.143 | 0.015 | 7.320E-23 | 0.008 | 0.014 | 5.898E-01 |
| rs7412 | 19 | 45412079 | T | C | 0.057 | -0.272 | 0.028 | 8.060E-22 | 0.130 | 0.026 | 9.330E-07 |

Supplementary Table 5 The differences between the various methods

| methods | differences |
| --- | --- |
| Inverse variance weighting with multiplicative random effects | Inverse variance weighting regressed instrument-outcome associations on instrument-exposure associations for the instruments, weighted by the inverse of the variance of the instrument-outcome association, with the intercept constrained to 0. Inverse variance weighting gives valid causal estimate when all instruments are valid instruments, i.e. instruments related to exposures; instrument independent of confounders, and exclusion restriction assumption. Inverse variance weighting will produce valid causal estimate in the presence of balanced horizontal pleiotropy and the instrument strength independent of direct effect (InSIDE) assumption is satisfied. |
| MR-Egger | MR-Egger method is similar to inverse variance weighting, but the intercept is not constrained to 0. As such, the MR-intercept test evaluates whether there is presence of overall horizontal pleiotropy. This method gives valid causal estimate even when all instruments are invalid. However, it relies on InSIDE assumption, which could be violated if the instruments are related to the confounders of exposure-outcome association. |
| Weighted median method | The weighted median method takes the weighted median of the Wald ratio as the causal estimate, weighted by the instrument-exposure association. Weighted median method gives valid causal estimate when more than 50% of the information is derived from valid instruments. |
| MR-PRESSO | MR-PRESSO provides a statistical test of (a) the presence of unbalanced horizontal pleiotropy (Global test), (b) generates a causal effect estimate corrected for unbalanced horizontal pleiotropy by outlier removal (Outlier corrected estimate), and (c) provides a statistical test comparing the estimate before and after the outlier removal (Distortion test). |
